# Maternal obesity, interpregnancy weight changes and congenital heart defects in the offspring: a nationwide cohort study

**DOI:** 10.1038/s41366-024-01531-5

**Published:** 2024-05-11

**Authors:** Gitte Hedermann, Paula L. Hedley, Kasper Gadsbøll, Ida N. Thagaard, Lone Krebs, Christian M. Hagen, Thorkild. I. A. Sørensen, Michael Christiansen, Charlotte K. Ekelund

**Affiliations:** 1 Department for Congenital Disorders, Danish National Biobank and Biomarkers, Statens Serum Institut, Copenhagen, Denmark; 2 Department of Obstetrics and Gynaecology, Copenhagen University Hospital Rigshospitalet, Copenhagen, Denmark; 3 Department of Obstetrics and Gynaecology, Slagelse Hospital, Slagelse, Denmark; 4 Department of Epidemiology, School of Public Health, University of Iowa, Iowa City, IA USA; 5 Centre of Foetal Medicine, Department of Obstetrics and Gynaecology, Copenhagen University Hospital Rigshospitalet, Copenhagen, Denmark; 6 Department of Obstetrics and Gynaecology, Nordsjaellands Hospital, Farum, Denmark; 7 Department of Obstetrics and Gynaecology, Copenhagen University Hospital Amager and Hvidovre Hospital, Copenhagen, Denmark; 8 Department of Clinical Medicine, University of Copenhagen, Copenhagen, Denmark; 9 Department of Public Health, Section of Epidemiology, University of Copenhagen, Copenhagen, Denmark; 10 The Novo Nordisk Foundation Center for Basic Metabolic Research, Faculty of Health and Medical Sciences, University of Copenhagen, Copenhagen, Denmark; 11 Department of Biomedical Sciences, University of Copenhagen, Copenhagen, Denmark

**Keywords:** Risk factors, Epidemiology

## Abstract

**Objective:**

To evaluate the association between maternal BMI and congenital heart defects (CHDs) in the offspring when including live births, stillbirths, aborted and terminated pregnancies and to investigate if maternal interpregnancy weight changes between the first and second pregnancy influences the risk of foetal CHDs.

**Methods:**

A nationwide cohort study of all singleton pregnancies in Denmark from 2008 to 2018. Data were retrieved from the Danish Foetal Medicine Database, which included both pre- and postnatal diagnoses of CHDs. Children or foetuses with chromosomal aberrations were excluded. Odds ratios were calculated with logistic regression models for CHDs overall, severe CHDs and five of the most prevalent subtypes of CHDs.

**Results:**

Of the 547 105 pregnancies included in the cohort, 5 442 had CHDs (1.0%). Risk of CHDs became gradually higher with higher maternal BMI; for BMI 25-29.9 kg/m^2^, adjusted odds ratio (aOR) 1.17 (95% CI 1.10-1.26), for BMI 30-34.9 kg/m^2^, aOR 1.21 (95% CI 1.09-1.33), for BMI 35-39.9 kg/m^2^, aOR 1.29 (95% CI 1.11-1.50) and for BMI ≥ 40 kg/m^2^, aOR 1.85 (95% CI 1.54-2.21). Data was adjusted for maternal age, smoking status and year of estimated due date. The same pattern was seen for the subgroup of severe CHDs. Among the atrioventricular septal defects (n = 231), an association with maternal BMI ≥ 30 kg/m^2^ was seen, OR 1.67 (95% CI 1.13-2.44). 109 654 women were identified with their first and second pregnancies in the cohort. Interpregnancy BMI change was associated with the risk of CHDs in the second pregnancy (BMI 2 to < 4 kg/m^2^: aOR 1.29, 95% CI 1.09-1.53; BMI ≥ 4 kg/m^2^: aOR 1.36, 95% CI 1.08-1.68).

**Conclusion:**

The risk of foetal CHDs became gradually higher with higher maternal BMI and interpregnancy weight increases above 2 BMI units were also associated with a higher risk of CHDs.

## Introduction

Obesity among women of reproductive age has been increasing over the last three decades [1]. Centers for Disease Control and Prevention estimated that 40% of women aged 20-39 years old in the United States had obesity (body mass index [BMI] ≥ 30 kg/m^2^) in 2017-2018 [2]. Maternal obesity is a risk factor for adverse pregnancy outcomes as well as for long-term health consequences for both the mother and child [3, 4]. Furthermore, maternal obesity is associated with a higher risk of having a child with congenital malformations [5].

Congenital heart defects (CHDs) remain the leading cause of infant death from congenital malformations in the United States [6]. Believed to be the most common congenital malformations, CHDs have a global prevalence of nine per 1000 live births with geographical differences [7]. The causes of CHDs are unknown in most cases but are associated with maternal age, chronic conditions, viral infections and foetal exposures to teratogenic drugs [8–11]. With improvements in genetic and genomic analytical techniques an increasing number of genetic associations/causes have been identified in up to 30% of the cases [12].

The association between maternal obesity and infants born with congenital malformations has been reported to include CHDs. However, none of the large studies have included the proportion of CHDs that are identified and terminated in pregnancy. During the last two decades, the prenatal identification of CHDs has increased dramatically, consequently, an analysis of the association between maternal risk factors and CHDs should include data on prenatally identified cases. Meta-analyses suggest a moderate association between maternal obesity (BMI ≥ 30 kg/m^2^) and CHDs in the offspring with an odds ratio (OR) 1.2 (95% CI 1.1-1.2) [13] or an OR 1.3 (95% CI 1.2-1.4) [14]. A recent systematic review on the topic demonstrated great heterogeneity among the studies concerning design, exposure definition, outcome definition, choice of covariates, and only populations of Northern European or Chinese descent were examined to a reasonable extent [15].

Some studies have found an association between maternal interpregnancy BMI changes and adverse pregnancy- and perinatal outcomes that were linearly related to the amount of weight gain [16, 17]. So far, a few small studies suggest that this might be relevant for certain congenital malformations (spina bifida, gastroschisis and oral cleft), [16, 18–20] however, no data is available in these studies for foetal CHDs. It is of great importance to identify any modifiable risk factors for CHDs. If weight gain, defined as interpregnancy BMI change, is associated with CHDs, this could be added to the aetiology of the association and the justification of preventive initiatives as to stress weight stability, and for some women weight loss.

The study hypothesized that high maternal BMI was associated with a higher risk of foetal CHDs when the study population comprised all CHDs found among live births, stillbirths, abortions and terminated pregnancies in Denmark. Furthermore, interpregnancy maternal BMI changes were hypothesized to influence the risk of foetal CHDs.

This study aims to assess the risk of foetal CHDs, severe CHDs or five of the most frequently identified subtypes of CHDs according to early-pregnancy BMI. The study also investigates if changes in maternal BMI from the beginning of the first pregnancy to the beginning of the second pregnancy were associated with the risk of CHDs in the second pregnancy.

## Materials and methods

This cohort study was performed on prospectively collected data retrieved from a nationwide cohort based on The Danish Foetal Medicine Database [21]. The Danish Foetal Medicine Database includes data on pregnancies with prenatal screening results from all obstetric and gynaecological departments in Denmark from January 1, 2008 [21]. All women in Denmark are offered a first-trimester screening for chromosomal abnormalities (gestational week 12) and a second-trimester anomaly scan, for which the uptake rate is high; 95% of pregnant women participate. The database does not include data on the outcomes of pregnancies before the first-trimester scan. The primary source of information is the local foetal medicine databases used nationwide in which sonographers and maternal-foetal medicine specialists add data from all examinations. The Danish Foetal Medicine Database includes data on maternal characteristics including weight and height, data from ultrasound examinations and pregnancy outcomes [21]. Furthermore, the database includes data from other Danish registers: the Danish Cytogenetic Central Register [22], the Danish National Patient Register [23], and the Danish Medical Birth Register [24]. All Danish residents are assigned a unique personal identification number enabling linkage of data between national registers and other data sources [25]. In Denmark, health care is free and it is standard practice to offer genetic testing by chorionic villus sampling or amniocentesis when a CHD is diagnosed prenatally [26]. The prenatal detection rate and accuracy of major CHD is high and the majority of parents opt for further testing [27, 28]. A gradual transition from conventional karyotyping to chromosomal microarray was observed throughout the study period. Postnatal genetic testing primarily by chromosomal microarray is performed in all children with syndromic suspicion. The Danish Foetal Medicine database is updated once a year with information on postnatal diagnosed congenital malformations and karyotypes [21]. The International Classification of Diseases, 10th revision code system (ICD-10) is used to code malformations in the foetus and the infant [21].

The cohort included singleton pregnancies in Denmark with an estimated due date, from an ultrasound scan, between June 1, 2008 and June 1, 2018. Each woman could have more than one pregnancy during the study period. Pregnancies with a foetus or child affected by a chromosomal aberration were excluded from the cohort. Only pregnant women with a registered weight and a height from 120 through 200 cm were included. Maternal weight was documented during the first antenatal appointment with the family doctor, occurring around gestational weeks 8-10, and was recorded as either self-reported prepregnancy weight or early-pregnancy weight. Maternal BMI was calculated as weight in kilograms divided by the square of the height in meters (kg/m^2^) and BMI values are reported in that unit. Extreme observations defined as BMI < 12 and BMI > 60 were excluded to avoid registration errors.

We identified foetuses and infants with CHDs (Table S1) by using either the prenatal or/and the postnatal diagnoses. In all live births, postnatal CHD diagnoses were considered the gold standard. The CHD diagnoses were defined by the European Surveillance of Congenital Anomalies (EUROCAT) [29] (Table S1). Severe CHDs include the following 17 diagnoses: truncus arteriosus, double outlet right ventricle, transposition of the great arteries (TGA), univentricular heart (UVH), atrioventricular septum defect (AVSD), Tetralogy of Fallot (ToF), pulmonary atresia, tricuspid valve stenosis, Ebstein anomaly, hypoplastic right heart syndrome, aortic valve stenosis, mitral valve stenosis, mitral insufficiency, hypoplastic left heart syndrome, coarctation of the aorta (CoA), aortic atresia, and total anomalous pulmonary venous return. Irrespective of the number of CHD diagnoses in a particular patient, the patient was only registered once as having CHDs. Furthermore, offspring with five of the most frequent subtypes of severe CHDs were identified (Table S2). These were ranked as defined by Lytzen et al. [27] with the most severe first (UVH > TGA > AVSD > CoA > ToF). If offspring had combinations of these subtypes, they were only registered once with the most severe diagnosis. ICD-10 codes for severe CHDs have been validated against hospital records with very good agreement in the Danish National Patient Register [30]. Prenatal diagnoses of 12 severe CHDs have been shown to have a very high diagnostic precision [28].

For the calculations of interpregnancy BMI changes, a sub-cohort including women with their first and second pregnancies was compiled. In the first pregnancy, they had to be nulliparous and they were not to have a fetus/child with CHDs. Parity information was not registered and therefore missing in the dataset from 2008-2011 corresponding to 25% of the pregnancies. If parity was recorded for a woman during subsequent pregnancies after 2011, it became feasible to determine her prior parity information, which was done. Maternal height was defined as the height registered in the first pregnancy. Interpregnancy BMI changes were calculated as the difference between BMI at the beginning of the first and BMI at the beginning of the second pregnancy. Differences were categorized into six groups < -2; -2 to < -1; -1 to < 1; 1 to < 2; 2 to < 4; and ≥4 BMI units. The category -1 to < 1 was defined as stable weight and used as a reference. A sensitivity analysis was done for women with a BMI ≥ 30 in the first pregnancy.

Associations between maternal BMI and offspring risk of CHDs were calculated as OR with 95% CI using logistic regression models. The models were adjusted for maternal age, maternal smoking status (yes/no), and year of estimated due date (1-year groups). The model was not adjusted for the possibility of a woman experiencing more than one pregnancy within the study period. Associations between interpregnancy BMI changes and risk of CHDs were calculated as OR with 95% CI using logistic regression models. Maternal BMI in the first pregnancy and maternal age at the second pregnancy were considered possible confounders and were adjusted for in the multivariate model. Statistical analyses were run in R version 4.2.1. Statens Serum Institut has approval from the Danish Data Protection Agency to conduct register-based studies, and the project has been approved (journal no. 19/03354 and 20/09279). The cohort study adhered to the STROBE guidelines. During the preparation of this work, the author used ChatGPT to improve language and readability. The authors reviewed and edited the content as needed and take full responsibility for the content of the publication.

## Results

**Fig. 1:**
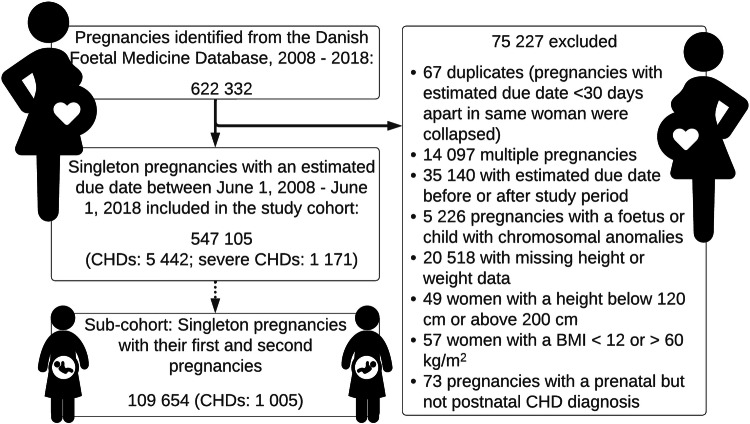
Flowchart. BMI body mass index, CHDs congenital heart defects.

The study cohort consisted of 547 105 singleton pregnancies with estimated due dates between June 1, 2008 and June 1, 2018 when pregnancies with chromosomal aberrations and missing data were excluded as detailed in Fig. 1. A total of 5 442 (1.0%) offspring had CHDs. Of these 1 171 were defined as severe CHDs (0.2%). Clinical and demographic data are available in Table 1. The study cohort comprised 534 406 live births (97.7%), 1 623 stillbirths (0.3%), 5 072 abortions or terminated pregnancies (0.9%), and 6 004 pregnancies with missing outcomes (1.1%). In total, 35% of the women had an early-pregnancy BMI ≥ 25, and 13% had obesity (BMI ≥ 30). The distribution of maternal BMI among the different groups and covariates can be seen in Table 1.

**Table 1:** Maternal, pregnancy and offspring characteristics in singleton pregnancies in Denmark 2008-2018.

Characteristic	Pregnancies without foetal CHDs, n = 541 663	Pregnancies with any foetal CHDs, n = 5 442	Pregnancies with severe foetal CHDs, n = 1 171
Maternal age	30.0 (26.0, 33.0)	30.0 (26.0, 33.0)	29.0 (26.0, 33.0)
BMI	23.0 (20.0, 26.0)	23.0 (21.0, 27.0)	23.0 (21.0, 27.0)
BMI groups, kg/m^2^			
<18.5	35 796 (6.6%)	366 (6.7%)	74 (6.3%)
18.5-24.9	318 481 (59%)	2 936 (54%)	624 (53%)
25-29.9	117 370 (22%)	1 287 (24%)	277 (24%)
30-34.9	45 925 (8.5%)	526 (9.7%)	118 (10%)
35-39.9	16 634 (3.1%)	202 (3.7%)	52 (4.4%)
≥40	7 457 (1.4%)	125 (2.3%)	26 (2.2%)
Caucasian ethnicity	492 478 (93%)	5 004 (94%)	1 082 (94%)
Missing maternal ethnicity	13 331 (2.5%)	126 (2.3%)	19 (1.6%)
Smoking in pregnancy	50 214 (9.3%)	622 (12%)	110 (9.5%)
Nulliparous	181 065 (45%)	2 026 (47%)	396 (44%)
Spontaneous conception	498 154 (93%)	4 900 (91%)	1,065 (92%)
Male sex	271 564 (51%)	2 744 (53%)	597 (63%)
Unknown offspring sex	12 388 (2.3%)	311 (5.7%)	216 (18%)
Delivery year			
2008-2011	193 046 (36%)	1 750 (32%)	413 (35%)
2012-2014	157 445 (29%)	1 613 (30%)	379 (32%)
2015-2018	191 172 (35%)	2 079 (38%)	379 (32%)
Outcome pregnancy			
Stillborn	1 582 (0.3%)	41 (0.8%)	26 (2.2%)
Live born	529 275 (98.8%)	5 131 (94.7%)	955 (82.6%)
Abortions incl. terminated pregnancies	4 827 (0.9%)	245 (4.5%)	175 (15.1%)
Unknown	5 979 (1.1%)	25 (0.5%)	15 (1.3%)

*CHDs* congenital heart defects, *IQR* interquartile range, *n* numbers.

Characteristics are given in either median (IQR) or n (%). Congenital heart defects are defined into subgroups “any” and “severe” according to the European Surveillance of Congenital Anomalies (diagnoses codes listed in Table S1).

Maternal overweight was associated significantly with higher risk of CHDs in the offspring increasing with higher maternal BMI: for BMI 25-29.9, adjusted OR (aOR) 1.17 (95% CI 1.10-1.26), for BMI 30-34.9, aOR 1.21 (95% CI 1.09-1.33), for BMI 35-39.9, aOR 1.29 (95% CI 1.11-1.50) and for BMI ≥ 40, aOR 1.85 (95% CI 1.54-2.21) compared to women with an early-pregnancy BMI 18.5-24.9 (Table 2). The predicted probability of CHDs by BMI as a continuous variable can be seen in Figure S1. Similar results were seen for maternal BMI and severe CHDs: for BMI 25-29.9, aOR 1.21 (95% CI 1.04-1.39), for BMI 30-34.9, aOR 1.29 (95% CI 1.05-1.57), for BMI 35-39.9, aOR 1.58 (95% CI 1.16-2.09) and for BMI ≥ 40, aOR 1.86 (95% CI 1.22-2.70) compared to women with an early-pregnancy BMI 18.5-24.9 (Table 2). Similar results were found when including live births only (for BMI 30-34.9, aOR 1.21 (95% CI 1.10-1.33), for BMI 35-39.9, aOR 1.30 (95% CI 1.12-1.50) and for BMI ≥ 40, aOR 1.84 (95% CI 1.53-2.20); data available in Table S3).

**Table 2:** Odds ratios of congenital heart defects by maternal BMI in 547 105 offspring (live births, stillbirths, abortions and terminated pregnancies), Denmark 2008–2018.

Maternal BMI (kg/m^2^)	All pregnancies, n = 547 105	Congenital heart defects, n = 5 442					Severe congenital heart defects, n = 1 171				
	Total	Prevalence [95% CI]	Crude OR	95% CI	aOR^a^	95% CI	Prevalence [95% CI]	Crude OR	95% CI	aOR^a^	95% CI
< 18.5	36 162	1.0% [0.91%, 1.12%]	1.11	0.99-1.24	1.08	0.96-1.20	0.2% [0.16%, 0.26%]	1.05	0.82-1.33	1.06	0.82-1.34
18.5–24.9	321 417	0.9% [0.88%, 0.95%]	1.00	ref	1.00	ref	0.2% [0.18%, 0.21%]	1.00	ref	1.00	ref
25–29.9	118 657	1.1% [1.03%, 1.15%]	1.19	1.11-1.27	1.17	1.10-1.26	0.2% [0.21%, 0.26%]	1.20	1.04-1.38	1.21	1.04-1.39
30–34.9	46 451	1.1% [1.04%, 1.23%]	1.24	1.13-1.36	1.21	1.09-1.33	0.3%[0.21%, 0.30%]	1.31	1.07-1.59	1.29	1.05-1.57
35–39.9	16 836	1.2% [1.04%, 1.38%]	1.32	1.14-1.52	1.29	1.11-1.50	0.3% [0.23%, 0.40%]	1.59	1.19-2.09	1.58	1.16-2.09
≥ 40	7 582	1.6% [1.37%, 1.96%]	1.82	1.51-2.17	1.85	1.54-2.21	0.3% [0.22%, 0.50%]	1.77	1.16-2.56	1.86	1.22-2.70

*aOR* adjusted odds ratios, *BMI* body mass index, *CHDs* congenital heart defects, *CI* confidence interval, *OR* odds ratio.

^a^aOR adjusted for maternal age, smoking status (yes/no), and year of estimated due date comparing foetal CHD risk in women with early-pregnancy BMI < 18.5 kg/m^2^ or BMI ≥ 25 kg/m^2^ with women with early-pregnancy BMI in the normal range (BMI 18.5-24.9 kg/m^2^).

**Fig. 2:**
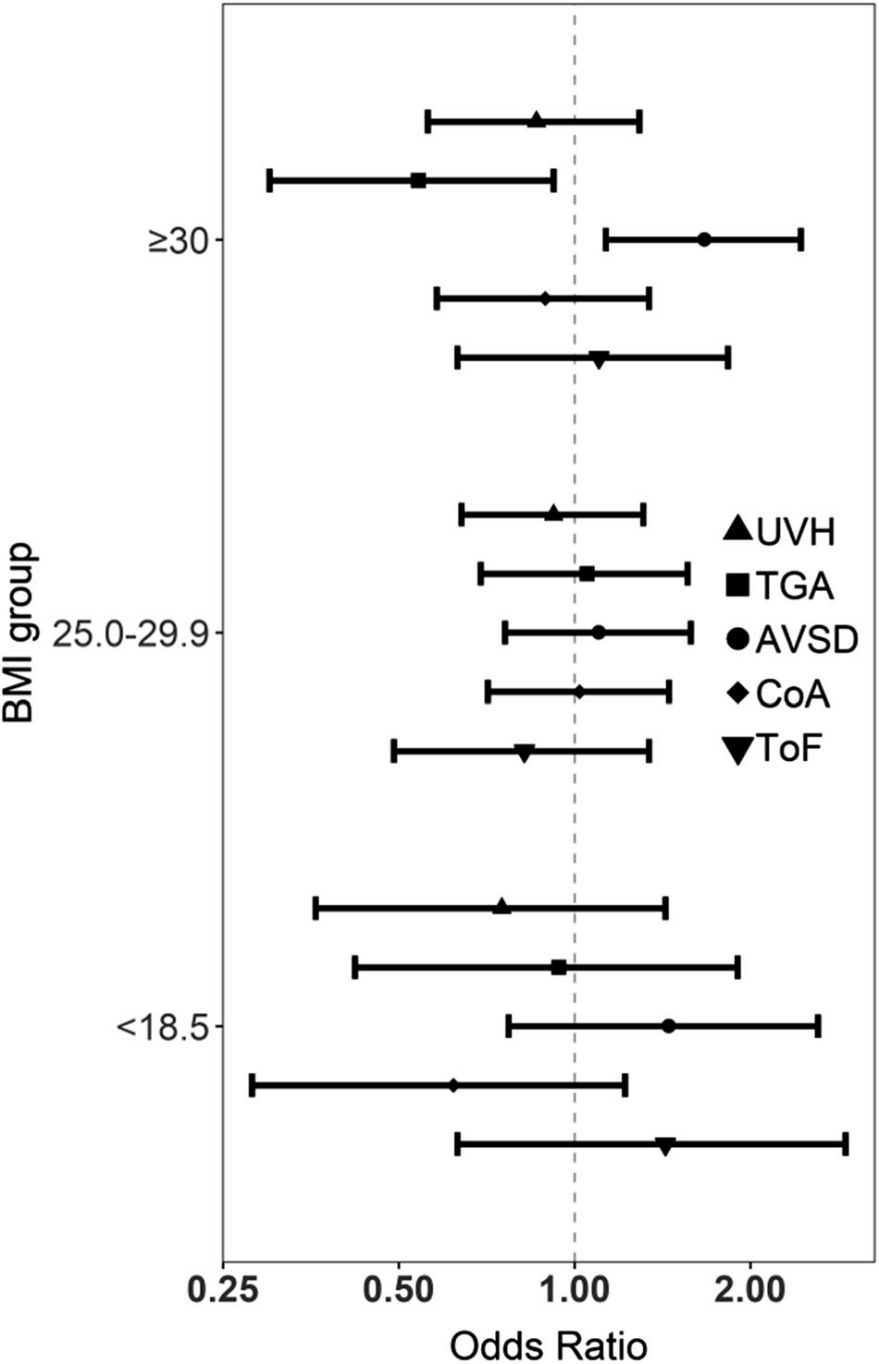
Crude odds ratios for five subtypes of congenital heart defects by maternal BMI, singleton pregnancies in Denmark 2008-2018. Crude OR with 95% CI for each CHD subtype per BMI group (underweight, BMI < 18.5; overweight. BMI 25-29.9; obesity, BMI ≥ 30). BMI 18.5-24.9 kg/m^2^ was considered as the normal range and used as a reference. AVSD atrioventricular septal defects, CHD congenital heart defect, CoA coarctation of the aorta, TGA transposition of the great arteries, ToF Tetralogy of Fallot, UVH univentricular heart.

The proportion of CHD cases with one of the five specific CHD diagnoses was as follows: univentricular heart (UVH; 4.2%), transposition of the great arteries (TGA; 2.8%), atrioventricular septum defect (AVSD; 4.2%), coarctation of the aorta (CoA; 4.1%), and Tetralogy of Fallot (ToF; 2.1%). The association between maternal BMI and these specific five CHD diagnoses were shown in Fig. 2. No significant associations were seen for UVH, TGA, CoA and ToF. However, maternal BMI ≥ 30 was positively associated with the risk of AVSD in the offspring (OR 1.67, 95% CI 1.13-2.44) (Fig. 2).

The sub-cohort included 109 654 women who had a first and second consecutive singleton pregnancy between June 1, 2008 and June 1, 2018 (Table 3). Mean BMI in first pregnancy was 23.4 vs. 24.0 in second pregnancy, the average BMI gain between the first and second pregnancies was 0.6 BMI units. In total, 1 005 had offspring with CHDs (0.9%) in their second pregnancy. The prevalence of CHDs in the second pregnancy became higher with increased weight gain (from ≥ 1 BMI unit to ≥ 4 BMI units) between pregnancies (0.9% to 1.2%). An increase in maternal BMI ≥ 2 BMI units between pregnancies was significantly associated with a higher risk of CHDs in the second pregnancy (aOR 1.29-1.36) when adjusted for maternal age in the second pregnancy and maternal BMI in the first pregnancy (Table 3, Fig. S2 and Table S4).

**Table 3:** Odds ratios of congenital heart defects in the second pregnancy by interpregnancy maternal BMI changes in 109 654 women with two consecutive singleton pregnancies, Denmark 2008-2018.

BMI change from 1st to 2nd pregnancy		1st pregnancy	2nd pregnancy	Risk of CHDs in 2nd pregnancy					
Units, kg/m2	No. of women with 1st and 2nd pregnancy	Mean BMI	Mean BMI	n	CHDs (%)	Crude OR	95% CI	aOR^a^	95% CI
<-2	4 466	28.5	24.2	46	1.0	1.25	0.91-1.68	1.05	0.75-1.43
-2 to <-1	6 870	24.6	22.6	50	0.7	0.88	0.65-1.17	0.85	0.63-1.13
-1 to <1	48 720	22.5	22.2	400	0.8	1.00	ref	1.00	ref
1 to <2	21 464	22.6	23.6	190	0.9	1.07	0.90-1.27	1.07	0.90-1.27
2 to <4	19 112	24.0	26.3	209	1.1	1.33	1.12-1.57	1.29	1.09-1.53
≥ 4	9 022	25.5	31.0	110	1.2	1.48	1.19-1.82	1.36	1.08-1.68
Total	109 654			1005					

*aOR* adjusted odds ratio, *BMI* body mass index, *CHDs* congenital heart defects, *CI* confidence interval, *n* number of women with their first pregnancy without CHDs and second pregnancy with CHDs in the offspring, *OR* odds ratio.

^a^aOR adjusted for maternal age in second pregnancy and early-pregnancy BMI at first pregnancy.

## Discussion

When including both pre- and postnatally diagnosed CHDs for all pregnancies, this study showed a dose-response association between high maternal BMI and risk of CHDs in the offspring. Additionally, a significant association was found between the risk of CHDs in the second pregnancy and interpregnancy BMI increase ≥ 2 BMI units between the first and second pregnancy.

This prospective nationwide cohort included 547 105 live births, stillbirths, abortions and terminated pregnancies in Denmark from 2008 to 2018 and showed that maternal overweight and obesity were significantly associated with a moderately higher risk of CHDs (aOR 1.17-1.85) and severe CHDs (aOR 1.21-1.86) in the offspring compared to women with a BMI in the normal range (BMI 18.5-24.9). The study validates previous findings of an association between high maternal BMI and risk of CHDs in the offspring [13, 14, 31, 32] and thereby rejects any hypothesis that this association is caused by lower prenatal detection rates of CHDs in pregnant women with obesity. Most other studies have been limited to live births [15, 31]. A large nationwide Swedish study with two million live-born children found an association between high maternal BMI and CHDs (BMI 30-34.9: OR 1.2, 95% CI 1.2–1.3; BMI ≥ 40: OR 1.6, 95% CI 1.4–1.8) [31]. When restricting our analysis to live births (Table S3), we found significant associations of a similar magnitude between maternal BMI ≥ 25 and foetal CHDs. Results from five specific subtypes of CHDs (UVH, TGA, AVSD, CoA and ToF) showed no significant association with maternal BMI except for AVSD (Fig. 2), which was significantly associated with increased risks when maternal BMI ≥ 30 (OR 1.67, 95% CI 1.13-2.44). Persson et al. found a non-significant association between maternal BMI and AVSD in women with BMI ≥ 30 [5], and the same pattern was observed in other studies [9, 33, 34]. The lack of statistical significance in other studies might be due to few cases in each BMI group or different study designs.

We examined the interpregnancy weight changes between the first and second pregnancies in 109 654 women. We found a higher risk of CHDs in the second pregnancy if the maternal weight increased ≥ 2 BMI units (Table 3). The investigations of the association between interpregnancy weight changes and the risk of foetal CHDs are sparse [16]. A few studies have looked at other congenital malformations and found a relative risk 2.3 for isolated cleft palate when maternal BMI increased ≥ 3 BMI units [20], an association between spina bifida and interpregnancy BMI gain [18], and a significant decrease (OR 0.62, 95% CI 0.42-0.94) for gastroschisis when maternal BMI increased with ≥ 3 BMI units [19].

### Clinical implications

Lifestyle interventions to reduce the risk of foetal CHDs have been suggested to be introduced before or between pregnancies [35]. Our findings partially support this by indicating that weight gain between pregnancies was associated with a higher risk of foetal CHDs compared to maintaining stable interpregnancy weight. However, this effect differed from the positive impact of weight reduction in women with obesity before pregnancy. In our sensitivity analysis including only women with BMI ≥ 30 in the first pregnancy (Table S4), we did not observe a reduction in the risk of foetal CHDs with weight loss between pregnancies for these women. Healthy People 2030 aims to reduce overweight and obesity by approx. 5% in the United States. About 13% of the Danish pregnant women had a BMI ≥ 30. If obesity were reduced by 5%, roughly 3 300 women with obesity should achieve normal weight. Assuming weight loss equated to a reduced risk of foetal CHDs from 1.25% to 1.00%, the potential reduction in CHD cases among their children could be from 41 to 33, preventing about 8 CHD cases over 10 years if obesity rates dropped by 5%. However, this would not impact the overall prevalence of CHDs. Nevertheless, weight loss might be significant for individual women. In a setting with prepregnancy counselling, it is still important to advise women about the importance of BMI as a risk factor for congenital malformations [5], obstetric and perinatal complications [3, 4].

### Research implications

Prenatal detection rates of congenital malformations decrease with increasing maternal BMI since image quality is lower in women with obesity [36, 37]. Consequently, studies only including live births could be biased towards a higher postnatal prevalence of CHDs in women with obesity if severe foetal CHDs were not diagnosed and possibly terminated during pregnancy (Table 1). Thus, this phenomenon may at least partly explain an association between high BMI and the prevalence of CHDs in live births [5]. Prenatal detection rates of CHDs have improved substantially in the last decades, and in countries with prenatal screening with high detection rates of CHDs as in Denmark [28], it is more important to include terminated pregnancies in prevalence and association studies as the rate of terminated pregnancies with the most severe CHDs likely will be higher as seen in Table 1.

This study confirms the association between high maternal BMI and risk of foetal CHDs. High maternal BMI is also associated with diabetes and hypertension, and both maternal conditions are associated with foetal CHDs [38, 39]. Knowledge about the aetiology is still limited [35] and future research should focus on combinations of other related maternal metabolic disorders linked to insulin resistance as suggested in a recent review [15].

### Strengths and Limitations

The strength of this study is the large size of the cohort and prospectively, nationwide data collection including prenatal information. Some limitations must be considered. The database did not include data on pregestational diabetes, which is known to be strongly associated with CHDs, and therefore this confounder was not included as a covariate in the analyses [15, 40]. Persson et al. excluded all women with pregestational diabetes and found a moderate association similar to the results of the present study [31]. Nor did the data include information about a family history of CHDs [41], maternal infections or teratogenic medicine intake in pregnancy that have been associated with higher risk of CHDs [8]. Since the study makes conclusions from register data, there is a risk of reporting bias and all CHD diagnoses are not validated against hospital records.

## Conclusion

This study found that the risk of foetal CHDs becomes gradually higher with higher maternal BMI when including live births, stillbirths, aborted and terminated pregnancies, and there was a significant association between interpregnancy maternal weight gain ≥ 2 BMI units and higher risk of foetal CHDs the second pregnancy.

### Supplementary information

Supplemental Material

## Data Availability

The dataset analysed for the current study is not available due to Danish legislation. However, researchers can apply access to the data from https://www.rkkp.dk/kvalitetsdatabaser/databaser/Dansk-Foetalmedicinsk-Database/.
