# Supplementary material for: Maternal obesity, interpregnancy weight changes and congenital heart defects in the offspring: a nationwide cohort study
Source: Int J Obes (Lond). 2024 May 11;48(8):1126–32. doi: 10.1038/s41366-024-01531-5 (PMC11281899; doi:10.1038/s41366-024-01531-5)

Supplementary Information – Maternal obesity, interpregnancy weight changes and congenital heart defects in the offspring: a nationwide cohort study

Table S1. Definition of congenital heart defects by ICD-codes

	ICD-10	Comments	Excluded diagnoses
CHDs (EUROCAT)	Q20-26	Exclude PDA with GA<37 weeks; Exclude peripheral pulmonary artery stenosis with GA<37 weeks	Q211C (Q2111: Patent or persistent foramen ovale), Q250 if GA<37 weeks (PDA), Q254E (Q2541: Persistent right aortic arch), Q256 if GA<37 weeks (Peripheral pulmonary artery stenosis), Q261 (Persistent left superior vena cava)
Severe CHDs (EUROCAT)	Q200	Truncus arteriosus	
	Q201	Double outlet right ventricle	
	Q203	Transposition of the great arteries (TGA)	
	Q204	Univentricular heart (UVH)	
	Q212	Atrioventricular septal defects (AVSD)	
	Q213	Tetralogy of Fallot (ToF)	
	Q220	Pulmonary atresia	
	Q224	Tricuspid valve stenosis	
	Q225	Ebstein anomaly	
	Q226	Hypoplastic right heart syndrome	

	Q230	Aortic valve stenosis	
	Q232	Mitral valve stenosis	
	Q233	Mitral insufficiency	
	Q234	Hypoplastic left heart syndrome	
	Q251	Coarctation of the aorta (CoA)	
	Q252	Interrupted aortic arch	
	Q262	Total anomalous pulmonary venous return	

Table S2. Definitions of five of the most frequently identified, perinatally as well as neonatally, subtypes of severe congenital heart defects

ICD-10	Diagnoses
DQ204<br>DQ234<br>DQ226	Univentricular heart (UVH) =<br>Hypoplastic left heart syndrome =<br>Hypoplastic right heart syndrome
DQ203	Transposition of the great arteries (TGA)
DQ212	Atrioventricular septal defects (AVSD)
DQ251	Coarctatio aortae (CoA)
DQ213	Tetralogy of Fallot (ToF)

Table S3. Live births and risk of congenital heart defects

	No CHDs	Congenital heart defects					Severe congenital heart defects				
	n = 529 275	n = 5 131					n = 955				
Maternal BMI (kg/m2)	Total	Total	Crude OR	95% CI	aOR*	95% CI	Total	Crude OR	95% CI	aOR*	95% CI
< 18.5	34 838	346	1.11	0.99,1.24	1.08	0.96,1.20	58	1.05	0.82,1.33	1.04	0.81,1.33
18.5 - 24.9	311 301	2 756	ref		ref		494	ref		ref	
25 - 29.9	114 739	1 214	1.19	1.11,1.27	1.18	1.10,1.26	233	1.20	1.04,1.38	1.21	1.05,1.40
30 - 34.9	44 873	496	1.24	1.13,1.36	1.21	1.10,1.33	98	1.31	1.07,1.59	1.28	1.04,1.57
35 - 39.9	16 234	197	1.32	1.14,1.52	1.30	1.12,1.50	49	1.59	1.19,2.09	1.58	1.17,2.10
≥ 40	7 290	122	1.82	1.51,2.17	1.84	1.53,2.20	23	1.77	1.16,2.56	1.87	1.23,2.71

Congenital heart defects defined by EUROCAT.

Abbreviations: aOR, adjusted odds ratio; BMI, body mass index; CHDs, congenital heart defects; CI, confidence interval; OR, odds ratio.

*aOR adjusted for maternal age, smoking status, and year of estimated due date comparing CHD risk in women with early-pregnancy BMI < 18.5 kg/m^2 or BMI ≥ 30 kg/m^2 with women with early-pregnancy normal BMI (18.5-24.9 kg/m^2).

Table S4. *Odds ratios of congenital heart defects in the second pregnancy by interpregnancy maternal BMI changes in women with a BMI ≥ 30 kg/m² in the first pregnancy and with two consecutive singleton pregnancies¤, Denmark 2008-2018*.

BMI change from 1st to 2nd pregnancy	Risk of CHDs in 2nd pregnancy				
Units, kg/m2	n	Crude OR	95% CI	aOR*	95% CI
<-2	1 546	1.34	0.77,2.31	1.46	0.82,2.55
-2 to <-1	884	0.96	0.43,1.93	1.06	0.47,2.15
-1 to <1	3 009	1.00	ref	1.00	ref
1 to <2	1 534	1.23	0.69,2.14	1.36	0.75,2.39
2 to <4	2 308	1.35	0.83,2.21	1.49	0.90,2.47
≥ 4	1 824	1.66	1.01,2.73	1.84	1.11,3.07
Total	11 105				

¤ Sensitivity analysis including only women with a BMI ≥ 30 kg/m² in the first pregnancy. * adjusted for maternal BMI in the first pregnancy and maternal age in the second pregnancy.

Figure S1. Predicted probability of fetal congenital heart defects by maternal BMI, Denmark 2008-2018.

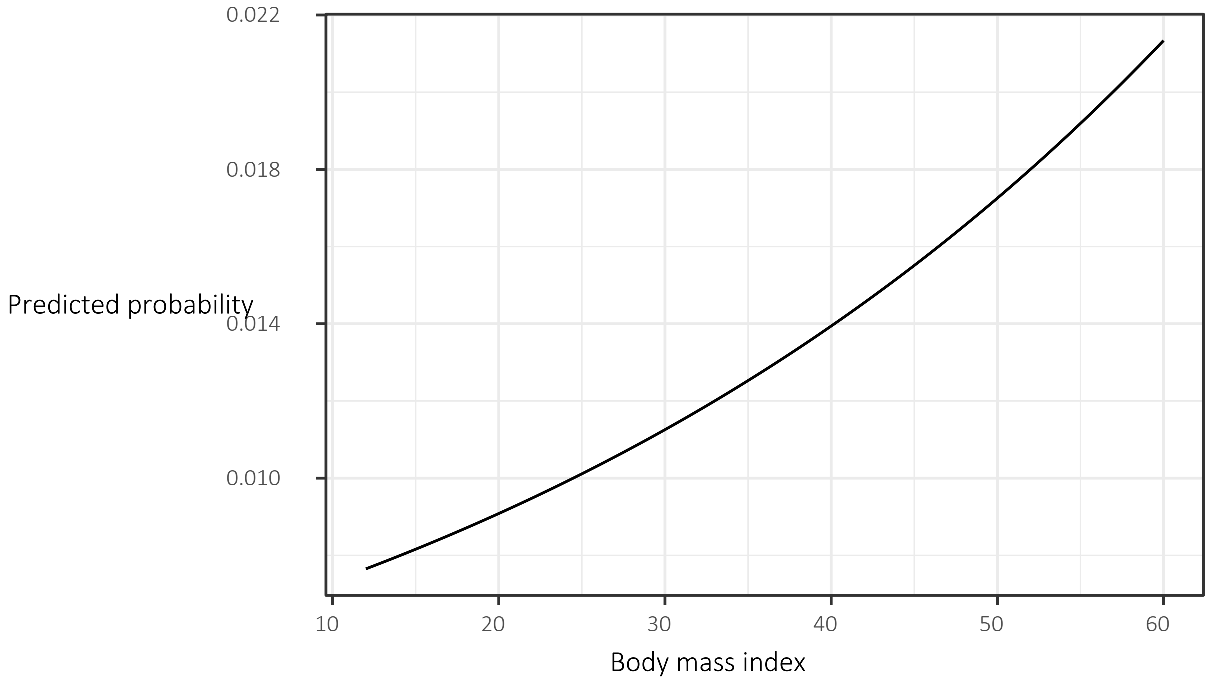

Figure S2. Predicted probability of fetal congenital heart defects by interpregnancy BMI changes in 109 654 women with two consecutive singleton pregnancies, Denmark 2008-2018.

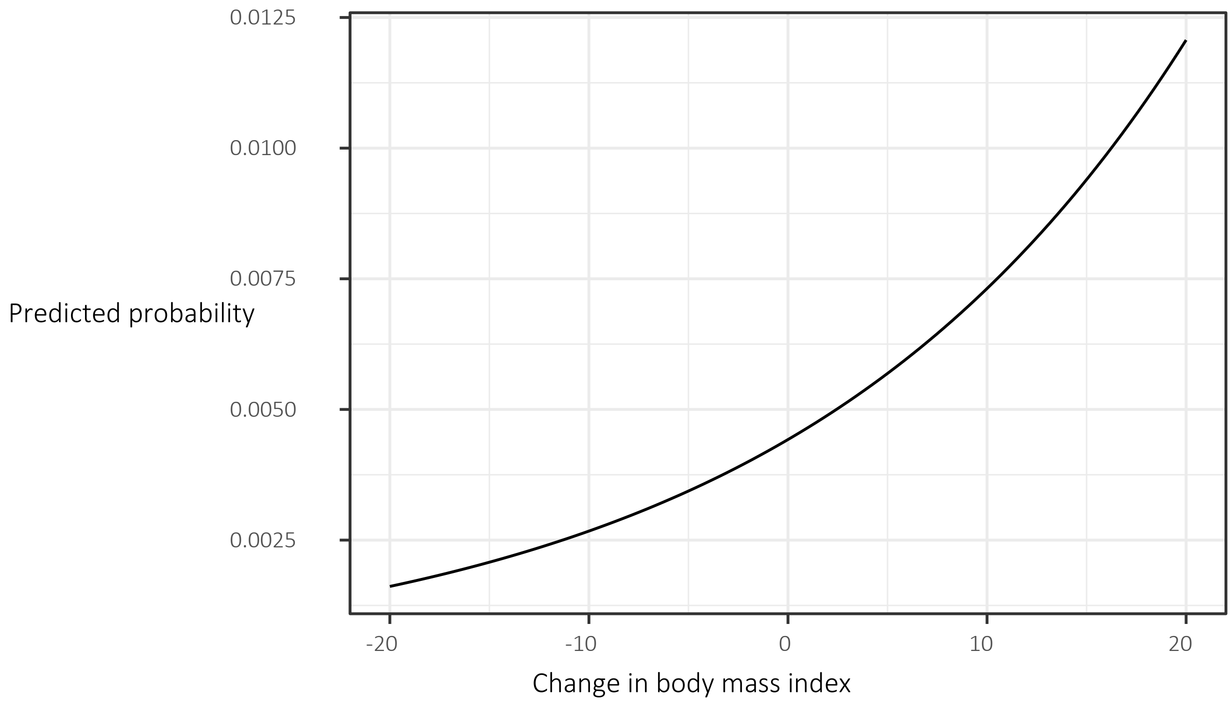

Supplement: Supplementary file 1 — Supplemental Material [file 41366_2024_1531_MOESM1_ESM.pdf]
